# Anti-Inflammatory and Anti-Oxidant Activity of *Portulaca oleracea* Extract on LPS-Induced Rat Lung Injury

**DOI:** 10.3390/molecules24010139

**Published:** 2019-01-01

**Authors:** Vafa Baradaran Rahimi, Hassan Rakhshandeh, Federica Raucci, Benedetta Buono, Reza Shirazinia, Alireza Samzadeh Kermani, Francesco Maione, Nicola Mascolo, Vahid Reza Askari

**Affiliations:** 1 Pharmacological Research Center of Medicinal Plants, Mashhad University of Medical Sciences, Mashhad 9177948564, Iran; baradaranv941@mums.ac.ir (V.B.R.); rakhshandehh@mums.ac.ir (H.R.); 2 Department of Pharmacology, Faculty of Medicine, Mashhad University of Medical Sciences, Mashhad 9177948564, Iran; 3 Department of Pharmacy, School of Medicine and Surgery, University of Naples Federico II, Via Domenico Montesano 49, 80131 Naples, Italy; federicaraucci@gmail.com (F.R.); bened.buono@gmail.com (B.B.); nicola.mascolo@unina.it (N.M.); 4 Department of Pharmacology, Faculty of Veterinary Medicine, University of Tehran, Tehran 1419963111, Iran; Rezashirazinia@ut.ac.ir; 5 Department of Chemistry, Faculty of Science, University of Zabol, Zabol 35856-98613, Iran; arsamzadeh@yahoo.com; 6 Neurogenic Inflammation Research Centre, Mashhad University of Medical Sciences, Mashhad 9177948564, Iran

**Keywords:** acute lung injury, *Portulaca oleracea*, inflammation

## Abstract

Acute lung injury (ALI) and acute respiratory distress syndrome (ARDS) are classified as two lung complications arising from various conditions such as sepsis, trauma, and lung inflammation. Previous studies have shown that the extract of the leaves of *Portulaca oleracea* (PO) possesses anti-inflammatory and anti-oxidant activities. In the present study, the effects of PO (50–200 mg/kg) and dexamethasone (Dexa; 1.5 mg/kg) on lipopolysaccharide (LPS)-induced ALI were investigated. Subsequentially, the lung wet/dry ratio; white blood cells (WBC); levels of nitric oxide (NO); myeloperoxidase (MPO); malondialdehyde (MDA); thiol groups formation; super oxide dismutase (SOD) and catalase (CAT) activities; and levels of interleukin (IL)-1β, tumor necrosis factor (TNF)-α, IL-6, IL-10, prostaglandin E2 (PGE_2_), and transforming growth factor (TGF)-β in the broncho alveolar lavage fluid (BALF) were evaluated in order to demonstrate the anti-oxidant and anti-inflammatory activity of PO. Our results show that PO suppresses lung inflammation by the reduction of IL-β, IL-6, TNF-α, PGE_2_, and TGF-β, as well as by the increase of IL-10 levels. We also found that PO improves the level of WBC, MPO, and MDA, as well as thiol group formation and SOD and CAT activities, compared with the LPS group. The results of our investigation also show that PO significantly decreased the lung wet/dry ratio as an index of interstitial edema. Taken together, our findings reveal that PO extract dose-dependently displays anti-oxidant and anti-inflammatory activity against LPS-induced rat ALI, paving the way for rational use of PO as a protective agent against lung-related inflammatory disease.

## 1. Introduction

Inflammation plays a dual protective and damaging role against cellular and tissue damages. Acute inflammation is usually considered a protective role to destroy and remove the noxious stimuli and injured tissues, thereby allowing the tissue repair. When this process becomes uncontrolled, another face of inflammation appears. In this regard, acute lung injury (ALI) and acute respiratory distress syndrome (ARDS) are known as two inflammatory lung complications with a high rate of morbidity and mortality [1,2]. These are characterized by severe pulmonary inflammation, massive recruitment of neutrophils and lymphocytes in interstitial tissue, edema, disruption of epithelial integrity, and the injury of lung parenchyma [3]. ALI and ARDS result from various diseases and pathological conditions such as trauma, pneumonia, sepsis, and endotoxemia [4,5].

Despite the great efforts to find new and/or most active pharmacological approaches for ALI/ARDS treatment and the discovery of the pathological factors, their mortality still presents a high rate (about of 40%) [6,7]. On this basis, the need for new therapeutic agents in the field of lung inflammatory-based diseases has prompted the investigation of several plant-based products for potential therapeutic application [8,9]. Portulaca oleracea (PO) belongs to the Portulacaceae family, commonly called qurfeh (Persia), purslane (the USA and Australia), pigweed (England), rigla (Egypt), pourpier (France), and Ma-Chi-Xian (China) [10]. This plant is commonly found in tropical and subtropical areas of the world, as well as in many regions of the United States, Mediterranean, and tropical Asian countries [11]. It has been used as a folk medicine in many countries for its febrifuge, antiseptic, and anthelmintic properties [12]. Congruently, it has been reported that PO exhibits great pharmacological properties including anti-oxidant [13], antibacterial [14], anti-ulcerogenic [15], anti-inflammatory [16], and wound-healing properties [17,18]. Indeed, recent studies have demonstrated that Oleracone, an alkaloid isolated from PO, attenuates inflammation induced by lipopolysaccharide (LPS) in RAW 264.7 macrophage cell lines [19] and that the extract of the leaves of PO possesses anti-inflammatory and immunomodulatory properties on Th1/Th2 lymphocytes profile [20]. It has also been reported that treatment of ovalbumin-sensitized rats with PO extract displays a modulation of lung inflammation and immune markers [21]. 

Lipopolysaccharide (LPS) is known as the predominant microbial inducer of inflammation processes accountable for the strong innate immune responses in ALI onset [5,22]. Alveolar epithelial cells (AECs) are the first cells faced by pathogenic microorganisms playing a central role in the beginning and progression of acute lung injury, followed by massive recruitment of neutrophils and lymphocytes [2,23]. Moreover, it has also been demonstrated that inflammation, due to inflammatory cyto-chemokines (in particular interleukin (IL)-6 and tumor necrosis factor (TNF)-α), plays an important role in lung injury onset [24,25] and the first innate immune response [26]. Therefore, in the present study, we aimed to investigate a new possible medication regarding the anti-inflammatory properties of PO for ALI using the LPS-induced animal model of ALI and lung inflammation. In the present study, we extended the previous observations about protective effects of PO, and shed new light on its anti-inflammatory and anti-oxidant mechanism of action.

## 2. Results

### 2.1. Effects of LPS and PO on Body and Absolute Organ Weights, and Lung Wet/Dry Ratio 

**Figure 1:**
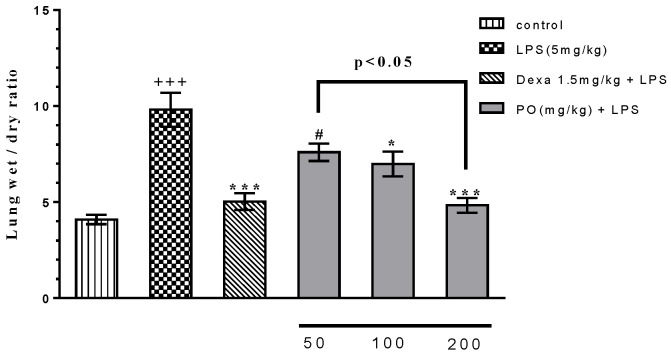
Effect of Portulaca oleracea (PO) extract on the lung wet/dry ratio in broncho alveolar lavage fluid (BALF). Data were presented as mean ± standard error of measurement (SEM) (*n* = 6). +++ *p* ≤ 0.001 compared with the control group, * *p* ≤ 0.05 and *** *p* ≤ 0.001 compared with the lipopolysaccharide (LPS) group, and # *p* ≤ 0.05 compared with the Dexa + LPS group.

LPS (5 mg/kg) significantly elevated the absolute organs weights (lung, *p* ≤ 0.001; liver *p* ≤ 0.01; and heart, *p* ≤ 0.001) compared with the control group (Table 1). PO at doses of 50 mg/kg (*p* ≤ 0.01), 100 mg/kg (*p* ≤ 0.001), and 200 mg/kg (*p* ≤ 0.001), as well as Dexa (1.5 mg/kg, *p* ≤ 0.001) significantly attenuated the absolute lung weight compared with the LPS group (Table 1). Moreover, PO (200 mg/kg) and Dexa (1.5 mg/kg) notably decreased the increased absolute weights of liver (*p* ≤ 0.01) and heart (*p* ≤ 0.001) compared with the LPS group (Table 1). LPS at 5 mg/kg significantly increased lung wet/dry ratio compared with the control group (Figure 1, *p* ≤ 0.001). PO at doses of 100 mg/kg (*p* ≤ 0.05) and 200 mg/kg (*p* ≤ 0.001) and Dexa (*p* ≤ 0.001) at 1.5 mg/kg significantly reverted the lung wet/dry ratio increase compared with the LPS group (Figure 1).

### 2.2. Effects of LPS and PO on Bronchoalveolar Lavage Fluid (BALF) Hematologic Indices

**Figure 2:**
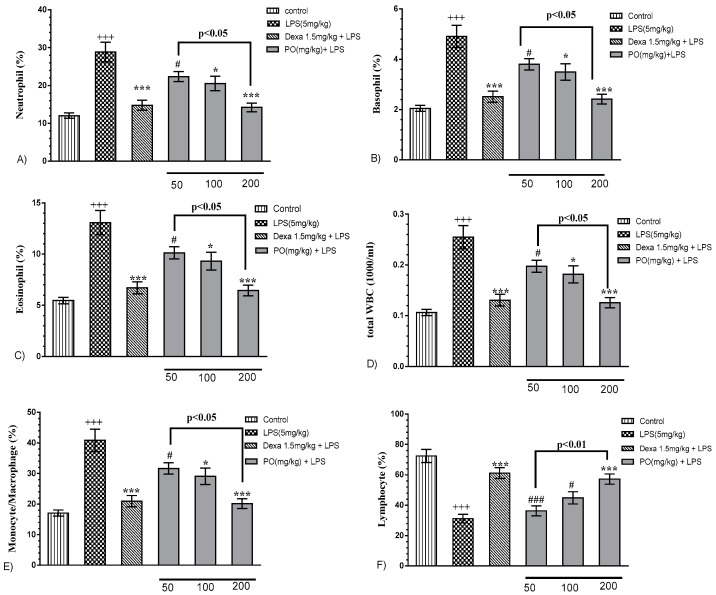
Effect of PO extract on hematologic indices (**A**) neutrophil, (**B**) basophil, (**C**) eosinophil, (**D**) total white blood cell, (**E**) monocyte/macrophage ratio, and (**F**) lymphocyte in BALF. Data were presented as mean ± SEM (*n* = 5). +++ *p* ≤ 0.001 compared with the control group, * *p* ≤ 0.05 and *** *p* ≤ 0.001 compared with the LPS-induced group and ^#^ *p* ≤ 0.05 and ^###^ *p* ≤ 0.001 compared with the Dexa + LPS group. WBC—white blood cells.

Our results revealed that LPS treatment significantly modified hematologic indices of neutrophil, basophil, eosinophil, and monocyte/macrophage, as well as total white blood cells (Figure 2A–E, *p* ≤ 0.001) and lymphocytes (Figure 2F, *p* ≤ 0.001). Notably, PO extract (100 and 200 mg/kg) and Dexa (1.5 mg/kg) markedly reverted hematologic indices (Figure 2A–E, *p* ≤ 0.05 to 0.001) and lymphocytes reduction (Figure 2F, *p* ≤ 0.001).

### 2.3. Effects of LPS and PO Extract on BALF Inflammatory Cytokines

**Figure 3:**
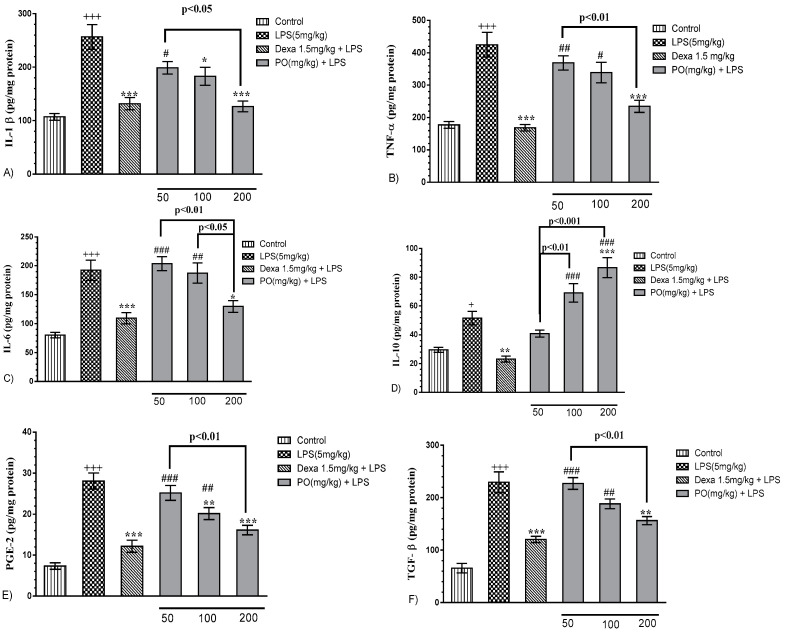
Effects of PO extract on inflammatory and anti-inflammatory biomarkers (**A**) interleukin (IL)-1β, (**B**) TNF-α, (**C**) IL-6, (**D**) IL-10, (**E**) PGE_2_, and (**F**) TGF-β in BALF. Data were presented as mean ± SEM (*n* = 7). ^+^ *p* ≤ 0.05 and ^+++^ *p* ≤ 0.001 compared with the control group; * *p* ≤ 0.05, ** *p* ≤ 0.01, and *** *p* ≤ 0.001 compared with the LPS-induced group; and ^#^ *p* ≤ 0.05, ^##^ *p* ≤ 0.01, and ^###^ *p* ≤ 0.001 compared with the Dexa + LPS group.

LPS significantly increased the production of inflammatory cytokines including IL-1β (Figure 3A, *p* ≤ 0.001), TNF-α (Figure 3B, *p* ≤ 0.001), IL-6 (Figure 3C, *p* ≤ 0.001), IL-10 (Figure 3D, *p* ≤ 0.05), PGE_2_ (Figure 3E, *p* ≤ 0.001), and TGF-β (Figure 3F, *p* ≤ 0.001) compared with the control group. Treatment with Dexa at 1.5mg/kg significantly decreased all measured parameters compared with the LPS group (Figure 3A–F, *p* ≤ 0.01 to 0.001 for all cases). Interestingly, PO at doses of 100 and 200 mg/kg significantly modulated the expression of IL-1β, TNF-α, IL-6, PGE_2_, and TGF-β, and the increase of increment in IL-10 level compared with the LPS group (Figure 3A–F, *p* ≤ 0.05 to 0.001 for all cases). 

### 2.4. Impact of PO on the BALF Oxidant/Antioxidant Status

**Figure 4:**
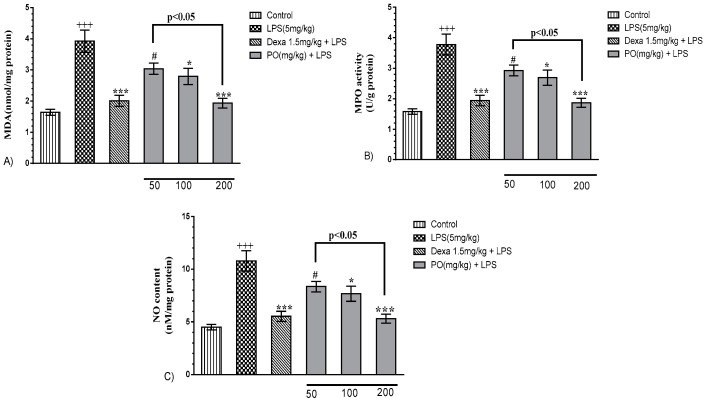
Effects of PO extract on oxidative indices: (**A**) malondialdehyde (MDA), (**B**) myeloperoxidase (MPO), and (**C**) NO in BALF and lung tissue. Data were presented as mean ± SEM (*n* = 7). ^+++^ *p* ≤ 0.001 compared with the control group, * *p* ≤ 0.05 and *** *p* ≤ 0.001 compared with the LPS-induced group, and ^#^ *p* ≤ 0.05 compared with the Dexa + LPS group.

**Figure 5:**
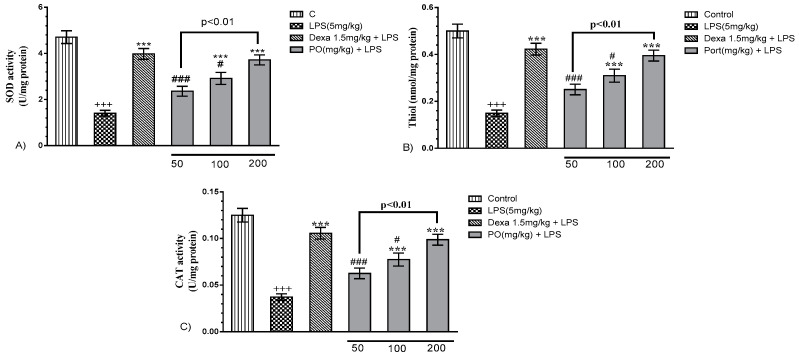
Effects of PO extract on anti-oxidative indices: (**A**) SOD, (**B**) thiol, and (**C**) catalase (CAT) in BALF and lung tissue. Data were presented as mean ± SEM (*n* = 6). ^+++^ *p* ≤ 0.001 compared with the control group, *** *p* ≤ 0.001 compared with the LPS-induced group, ^#^ *p* ≤ 0.05 and ^###^ *p* ≤ 0.001 compared with the Dexa + LPS group.

LPS notably increased malondialdehyde (MDA), myeloperoxidase (MPO), and nitric oxide (NO) levels, and contextually decreased super oxide dismutase SOD and catalase (CAT) activity, as well as thiol content, compared with the control group (Figure 4A–C, *p* ≤ 0.001). However, PO (100 and 200 mg/kg) and Dexa (1.5 mg/kg) groups significantly decreased MDA, MPO, and NO levels (Figure 4A–C, *p* ≤ 0.05 to 0.001) and reverted oxidant/anti-oxidant status that resulted in a significant increase in the levels of SOD (*p* ≤ 0.001) and catalase (*p* ≤ 0.001) activity, as well as total thiol content (*p* ≤ 0.001) (Figure 5). 

### 2.5. Characteristics of PO Hydro-Ethanolic Extract 

**Figure 6:**
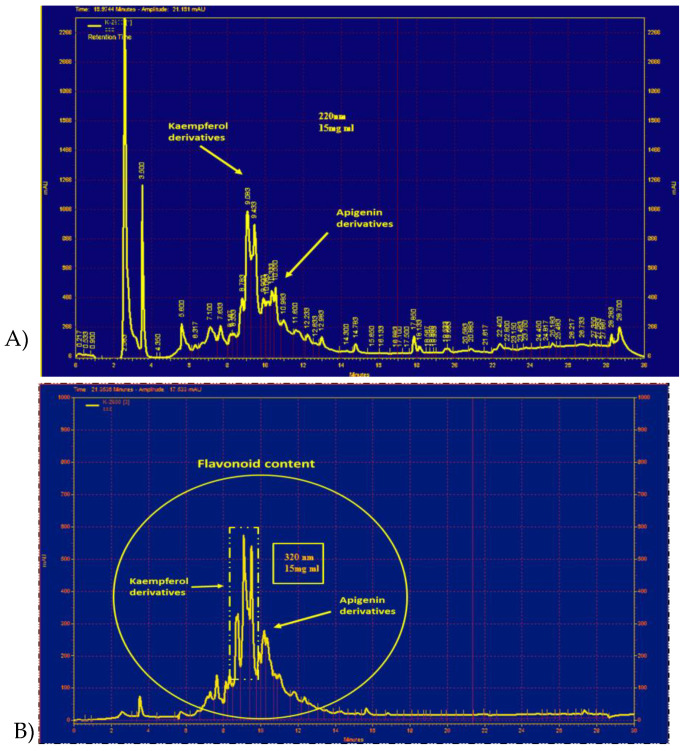
HPLC fingerprint of the hydro-ethanolic extract of *P. oleracea* at 220 nm (**A**) and 320 nm (**B**) (reprinted with permission from Ref. [20]).

HPLC peaks from PO extract chromatogram appeared at 320 nm (Figure 6B). Using the H-NMR method, we also detected the peaks at 220 and 320 nm between 8 and 12 min ascribed to kaempferol and apigenin derivatives, respectively (Figure 6A,B).

## 3. Discussion

LPS, as a gram-negative bacterial constituent, is a well-known and established cause of adventitious pneumonia from the community or hospital patients, and Toll-like receptor-4 (TLR4) activation by this stimuli is a reasonable way for activating the innate immunity against gram-negative pathogen/s [27,28]. It has been demonstrated that LPS leads to pulmonary inflammation such as ALI, which occurs after 4–48 h of pathogen exposure [29,30,31]. For this reason, previous studies have reported that LPS stimulation is a standard model for inducing experimental ALI and ARDS [32,33]. 

Inflammation is considered the major response to infections or injuries. However, uncontrolled prolongation of the inflammatory repertoire may lead to tissue damage and healing [24,28]. LPS activated macrophages produce inflammatory mediators such as TNF-α, PGE_2_, IL-1β, and IL-6 [34,35,36], which play an important role in self-sustaining inflammatory conditions [37]. Therefore, suppressing the prolonged and/or chronic lung inflammation induced by LPS could represent a potential curative aspect for lung injury [38,39]. 

*Portulaca oleracea* is a medicinal plant widely used in traditional medicine [40]. Nowadays, many studies have revealed its valuable pharmacologic properties including anti-inflammatory, anti-oxidant, anti-microbial, and neuroprotective [41,42,43,44,45,46,47,48]. PGE_2_ is a bioactive lipid associating with inflammation and cancer. Its synthesis is originated by phospholipases (PLAs) through liberating free fatty acids from membrane, including arachidonic acid (AA) [49]. There are several studies on the notion of the contribution of the dysregulation in the levels of arachidonic acid, and PGE_2_ synthesis or degradation have been associated with a number of inflammatory diseases [49,50,51]. In the current study, we demonstrated that LPS significantly increases the levels of PGE_2_ and other inflammatory cytokines in the BALF compared with the control group. Previous studies also described that an increase in the level of PGE_2_ promotes the activation of both the innate and adaptive immunity including macrophage and Th_17_ cells, a subset of CD4^+^ helper T cells, by the production of interleukin-17 (IL-17) [49,50,51]. Our results showed that PO suppresses lung inflammation by the reduction of IL-1β, IL-6, TNF-α, PGE_2_, and TGF-β, as well as by the increase of IL-10 levels. These results are in accordance with previous studies that reported anti-inflammatory effects of PO on RAW 264.7 macrophage cell line and human umbilical vein endothelial cells (HUVECs) treated with LPS [16,52]. In contrast, it has been reported that PO extract inhibits TNF-α-induced adhesion of HL-60 cells to HUVECs and mRNA expression of IL-8 and monocyte chemoattractant protein (MCP)-1 [12]. It has also been suggested that inhibition of inflammation by PO may be partly because of modulation of nuclear factor-kappa B (NF-κB) signaling pathway and decrement of p65 nuclear accumulation [53]. Indeed, all these pieces of evidence further support and validate the anti-inflammatory potential of *Portulaca oleracea* in other inflammatory-based illness such as colitis [54], peripheral pain [16], and liver injury [55]. Previous studies demonstrated that the levels of inflammatory (TNF-α and IL-1β) and anti-inflammatory (IL-10) cytokines are impressed by both transcription factors NF-κB and nuclear factor erythroid 2-related factor 2 (Nrf2), respectively [28,56,57]. As one of the current study’s limitations, we did not evaluate changes in gene expression and/or activity of certain transcription factors related to the inflammatory and antioxidant response such as NFκB and Nrf2, respectively. 

TLR-4, as the main receptor for LPS, possesses an essential role in the pathogenesis of ALI, also supported by its localization on airway vascular endothelial and epithelial cells [58]. Accordingly, Askari and coworkers [20] have shown that PO extract had modulatory effects on imbalanced lymphocytes through the reduction of inflammatory cytokines and increased expression of IL-10 may be correlated to TLR-4 modulation. LPS-induced lung injury is also characterized by neutrophils infiltration in lung tissues that occurs and is driven by the massive cytokines production [2,59,60]. Interestingly, we found that PO extract improves the level of WBC in BALF samples, as well as the level of MPO, compared with the LPS group as an index of a significative reduction of neutrophil recruitment and activity. Contextually, in ALI/ARDS onset, lung edema appears as a consequence of microvascular leakage associated with endothelial injury [61,62]. The results of our study showed that PO significantly decreased the lung wet/dry ratio as an index of interstitial edema. In addition, we observed that PO markedly prevented MDA and NO production as well as propagated thiol, catalase, and SOD in BALF. Lipid peroxidation is a condition under which free radicals attack lipids containing carbon–carbon double bonds leading to cell and organ damage [63,64,65]. The main bio-products produced in this process are MDA and SOD, widely used as lipid peroxidation index and as markers of lipid peroxidation in ALI/ARDS [66]. Furthermore, it has been suggested that NO and inducible nitric oxide synthase (iNOS) lead to oxidative stress and endothelial damage [63,64,65,67]. Therefore, inhibition of iNOS and reduction of NO content could be beneficial in these pathologies [68]. Free radicals are highly reactive species capable of destructing DNA, proteins, carbohydrates, and lipids, structurally leading to cell damage or apoptosis. Exogenous anti-oxidant enzymes such as catalase, SOD, and thiol groups protect cells against oxidative stress damages induced by free radicals [69,70,71,72,73]. It could be concluded that the significant modulation of SOD and catalase activities and of the level of thiol groups after PO treatments improve the lung antioxidant status in an LPS-induced rat lung injury. 

The anti-oxidant and anti-inflammatory properties of this plant extract may be the result of the considerable amounts of anti-oxidant compounds including gallotannins, omega-3 fatty acids, ascorbic acid, α-tocopherols, kaempferol, quercetin, and apigenin [16,74,75], and because of the presence of flavonoids such as kaempferol and apigenin derivatives found in our chemical characterization. Accordingly, Chen et al. reported that aqueous extract of PO alleviated high fat diet-elicited oxidative stress including blood and liver anti-oxidant enzymatic systems by the modulation of leptin and liver peroxisome proliferator-activated receptor (PPAR)-γ [13,28]. Furthermore, it has been reported that aqueous extract of PO exhibits cytoprotective effects on 2,2′-azobis hydrochloride-induced hemolytic injuries of red blood cells (RBCs) [76]. 

## 4. Materials and Methods

### 4.1. Chemicals and Reagents

LPS (Escherichia coli 055: B5) was obtained from Sigma (St. Louis, MO, USA). Myeloperoxidase (MPO) assay kit, IL-1β, TNF-α, IL-6, IL-10, PGE_2_, and TGF-β enzyme-linked immunosorbent assay (ELISA) kit were provided by eBioscience (San Diego, CA, USA). All other reagents were of analytical grade.

### 4.2. Preparation of PO Extract

PO extract was prepared according to our previously characterized and standardized method [20]. In brief, the hydroethanolic extract was made using the macerated method with 70% *w*/*w* ethanol for 72 h at room temperature (RT). The yield of the dried extract was 21% *w/w* in the ratio of dried powder, and kept in −20 °C until use. For the characterization of PO extract, we used a liquid chromatography system comprising of a 510 Waters pump (Waters Association, Milford, MA, U.S.A.), a variable wavelength (model 486 Waters UV detector), and a Waters sample injection system (U6K system). The mobile phase was formed by a mixture of methanol:acetonitrile/tetrahydrofuran/0.5% glacial acetic acid (5:3:18:74). This phase was filtered under vacuum, then degassed, and finally pumped through the Novapak C18 column (150 × 3.9 mm i.d.) at a flow rate of 1.0 mL/min. The chromatograms were recorded at 220 and 320 nm [62] and interpreted by Gloexir Pars ^®^ Company, Mashhad, Iran. For total flavonoid content, we have followed the procedure previously adopted in our group [63,64]. Briefly, 5 mL of 2% aluminum trichloride (AlCl_3_) in methanol was mixed with the same volume of PO extract, incubated for 15 min at RT, and then measured at 510 nm using a MultiSpec UV-vis spectrophotometer (Shimadzu, Tokyo, Japan). The total flavonoid content was measured based on the standard curve of different flavonoids at a range of 0–50 µg/mL. 

### 4.3. Animals and Husbandry

Adult male Sprague–Dawley rats (weighing 220 ± 30 g) were provided by the animal laboratory of Faculty of Medicine, Mashhad University of Medical Sciences (Mashhad, Iran), and kept in an animal care facility under controlled temperature and humidity, and on a 12 h/12 h light/dark cycle, with ad libitum access to water and a standard laboratory chow diet. All experimental procedures were carried out in compliance with local and national law and policies (Grant No. 971254 of Ethical Committee of National Institute for Medical Research Development, Iran). All procedures were carried out to minimize the number of animals (*n* = 5–7 per group) and their suffering. 

### 4.4. Experimental Protocol

Animals were randomly divided into six groups of 5–7 rats: (I) control group (saline); (II) LPS group (5 mg/kg); (III) LPS + Dexamethasone group (Dexa, 1.5 mg/kg); (IV) LPS + PO (50 mg/kg) group; (V) LPS + PO (100 mg/kg) group; and (VI) LPS + PO (200 mg/kg) group. LPS was administered using intraperitoneal (i.p.) injection to induce acute lung injury. PO and Dexa were administrated by oral gavage (po) 1 h before LPS injection. Dose adjustment was performed according to our preliminary study. Rats were sacrificed, and samples were collected at 4 h after LPS administration [61].

### 4.5. Absolute Organ Weight and Lung Wet/dry Weight Ratio

The whole body and absolute lung, heart, and liver weights were recorded. The measurement of water content of lungs was also carried out by assessing the wet/dry weight quantitative ratio of lung tissues. Briefly, the right lung inferior lobe was dissected and weighed to supply the ‘wet’ weight. The lung was then dried at −80 °C for 72 h to get the ‘dry’ weight. Finally, the wet/dry ratio was calculated as wet/dry ratio = wet weight/dry weight.

### 4.6. Broncho-Alveolar Lavage Fluid (BALF) Preparation

As previously described [64], animals were anesthetized with urethane (50 mg/kg, i.p.) at the end of the experimental protocol. Successively, the chest was opened and lungs and trachea were dissected and washed with distilled water. Lungs were lavaged with 1 mL saline five times at controlled RT through the cannulated trachea. The collected BALF was centrifuged at 2500 g for 10 min and the obtained supernatant was stored at −80 °C for the subsequent analysis.

### 4.7. Measurement of Total and Differential White Blood Cells (WBC) in BALF

Briefly, 1 mL of BALF was stained with Turk solution (1 mL glacial acetic acid, 1 mL of gentian violet solution 1%, and 100 mL distilled water) and counted by Neubauer chamber. Differential cell analysis was carried out by the aim of a light microscope as previously described [77]. WBC and differential WBC were determined via light microscope according to morphological criteria and staining.

### 4.8. Oxidant-Antioxidant Assessment in BALF

For measurement of malondialdehyde (MDA) concentration, the sample was refluxed with a solution of HCl and TBA (thiobarbituric acid). Then, 2 mL of this solution was added to 1 mL of BALF and heated in a water bath at 50 °C for 40 min to dissolve TBA. After cooling and reaching RT, it was centrifuged at 1000 rpm for 10 min. Afterward, the absorbance was read at 535 nm and then the MDA concentration was calculated based on the following equation: C (M) = A/1.65 × 10^5^, where C indicated the concentration and A the absorbance [24,78]. To determine the thiol groups, the DTNB method (2, 2′-dinitro-5, 5′-dithiodibenzoic acid) was used. This reagent reacts with SH (thiol) groups and produces a yellow complex that has an absorbance peak at 412 nm and has a molar absorption coefficient of 13.6 mM^−1^cm^−1^. Then, 1 mL of Tris-EDTA buffer (pH 8.6) was added to 50 μL BALF and sample absorbance was read at 412 nm against Tris-EDTA buffer alone (A1). A total of 20 μL of DTNB reagents (10 nM in methanol) was added to the mixture and the samples were kept at RT for 15 min, after which the absorbance was read again (A2). The absorbance of DTNB reagent was also read as a blank (B) [78]. Total thiol concentration expressed as nmol/mg protein was calculated with the following equation: (A2 − A1 − B) × 1.07/0.05 × 13.6. Catalase (CAT) activity was measured based on its ability to decompose hydrogen peroxide (H_2_O_2_) by decreasing adsorption in a 240 nm absorption spectrum. For this purpose, 30 mM hydrogen peroxide was used as a substrate and 50 mM phosphate buffer with pH = 7 as substrate substitute in blank solution. The measuring solution contained a suitable volume of sample and a solution of hydrogen peroxide. The reaction was started by adding hydrogen peroxide and reduced absorption by spectrophotometer at 240 nm for 3 min. Each catalase activity unit was defined as the hydrogen peroxide μmol consumed per mg of protein [78]. Measurement of superoxide dismutase enzyme activity was performed based on previously described methods with slight modifications [63,64,79,80,81]. The method is based on the pyrogallol auto-oxidation and the inhibition of superoxide-dependent reduction of the tetrazolium dye, MTT (3-(4,5-dimethylthiazol-2-yl)-2,5-diphenyltetrazolium bromide) to its formazan. The reaction was stopped by adding dimethyl sulfoxide (DMSO), and then performed with a microtiter plate reader at 570 nm. A unit of SOD was defined as the amount of enzyme needed to control MTT reduction up to 50%.

### 4.9. Enzyme-Linked Immunosorbent Assay (ELISA) Assay

The levels of TNF-α, IL-6, IL-1β, IL-10, PGE_2_, and TGF-β in the supernatants of obtained BALF were measured using commercially available enzyme-linked immunosorbent assay kit (ELISA kits, eBioscience Co., San Diego, CA, USA) according to the manufacturer instructions. Briefly, 100 μL of BALF supernatants, diluted standards, quality controls, and dilution buffer (blank) were applied on a pre-coated plate with the monoclonal antibody for 2 h. After washing, 100 μL of biotin-labeled antibody was added and incubation continued for 1 h. The plate was washed and 100 μL of the streptavidin–HRP conjugate was added and the plate was incubated for a further 30 min period in the dark. The addition of 100 μL of the substrate and stop solution represented the last steps before the reading of absorbance (measured at 450 nm) on a microplate reader [82].

### 4.10. Myeloperoxidase Assay

Leukocyte myeloperoxidase (MPO) activity was assessed by measuring the H_2_O_2_-dependent oxidation of 3,3′,5,5′-tetramethylbenzidine (TMB) as previously reported [82]. Aliquots of 20 μL of BALF were incubated with 160 μL of TMB and 20 μL of H_2_O_2_ (in 80 mM phosphate buffer, pH 5.4) in 96-well plates. Plates were incubated for 5 min at RT and optical density was read at 620 nm using a plate-reader (Biorad, Italy). The assay was performed in duplicates and normalized for protein content [82].

### 4.11. Statistical Analysis

The results obtained were expressed as the mean ± SEM. Normality test was carried out based on Kolmogorov–Smirnov. After passing the tests, statistical analysis was performed using one-way analysis of variance (ANOVA) followed by Bonferroni or Dunnett’s post-test when comparing more than two groups. Student’s t-test was used in cases where two groups were compared. Statistical analysis was performed using GraphPad Prism 6.0 software (San Diego, CA, USA). Data were considered statistically significant when a value of *p* ≤ 0.05 was achieved. The data and statistical analysis comply with the recommendations on experimental design, analysis [83], and data sharing and presentation in preclinical pharmacology [84,85].

## 5. Conclusions

These studies are in accordance with our findings that demonstrate the protective effects of PO on LPS-induced acute rat lung injury, paving the way for rational use of this plant extract in lung-related inflammatory diseases, as well as in those characterized by an increase of free radicals and oxidative reactive products.

## Figures and Tables

**Table 1:** Body and absolute organs weights for different groups.

	C	LPS	Dexa	PO 50 mg/kg	PO 100 mg/kg	PO 200 mg/kg
Body weight (g)	220 ± 31	218 ± 22	208 ± 19	215 ± 21	223 ± 22	231 ± 19
Lung (g)	1.05 ± 0.08 ***	2.78 ± 0.15	1.28 ± 0.07 ***	1.87 ± 0.14 **	1.61 ± 0.09 ***	1.23 ± 0.11 ***
Liver (g)	5.41 ± 1.21 **	7.94 ± 1.24	5.77 ± 1.06 **	8.01 ± 1.28	7.23 ± 1.18	5.69 ± 1.13 **
Heart (g)	0.86 ± 0.08 ***	1.21 ± 0.09	0.89 ± 0.12 ***	1.17 ± 0.07	1.05 ± 0.17	0.83 ± 0.12 ***

Data are presented as means ± SD (*n* = 6). ** *p* ≤ 0.01 and *** *p* ≤ 0.001. PO—Portulaca oleracea.
