# Correction: Baradaran Rahimi et al. Anti-Inflammatory and Anti-Oxidant Activity of *Portulaca oleracea* Extract on LPS-Induced Rat Lung Injury. *Molecules* 2019, *24*, 139

**DOI:** 10.3390/molecules30030443

**Published:** 2025-01-21

**Authors:** Vafa Baradaran Rahimi, Hassan Rakhshandeh, Federica Raucci, Benedetta Buono, Reza Shirazinia, Alireza Samzadeh Kermani, Francesco Maione, Nicola Mascolo, Vahid Reza Askari

**Affiliations:** 1 Pharmacological Research Center of Medicinal Plants, Mashhad University of Medical Sciences, Mashhad 9177948564, Iran; baradaranv941@mums.ac.ir (V.B.R.); rakhshandehh@mums.ac.ir (H.R.); 2 Department of Pharmacology, Faculty of Medicine, Mashhad University of Medical Sciences, Mashhad 9177948564, Iran; 3 Department of Pharmacy, School of Medicine and Surgery, University of Naples Federico II, Via Domenico Montesano 49, 80131 Naples, Italy; federicaraucci@gmail.com (F.R.); bened.buono@gmail.com (B.B.); nicola.mascolo@unina.it (N.M.); 4 Department of Pharmacology, Faculty of Veterinary Medicine, University of Tehran, Tehran 1419963111, Iran; Rezashirazinia@ut.ac.ir; 5 Department of Chemistry, Faculty of Science, University of Zabol, Zabol 35856-98613, Iran; arsamzadeh@yahoo.com; 6 Neurogenic Inflammation Research Centre, Mashhad University of Medical Sciences, Mashhad 9177948564, Iran

In the original publication [1], there was a mistake in the caption for Figure 6 as published. The authors had unintentionally re-used chromatograms from a previous publication but neglected to properly cite the previous work. However, the authors referenced the previous publication as Ref. [20] in the main text of the original publication [1]. The correct caption appears below. The authors state that the scientific conclusions are unaffected. This correction was approved by the Academic Editor. The original publication has also been updated.

**Figure 6.** HPLC fingerprint of the hydro-ethanolic extract of *P. oleracea* at 220 nm (**A**) and 320 nm (**B**) (reprinted with permission from Ref. [20]).
